# Biomedical Sensors for Functional Mapping: Techniques, Methods, Experimental and Medical Applications

**DOI:** 10.3390/s23167063

**Published:** 2023-08-10

**Authors:** Alfonso Mastropietro, Massimo Walter Rivolta, Alessandro Scano

**Affiliations:** 1 Istituto di Tecnologie Biomediche, Consiglio Nazionale delle Ricerche, 20054 Segrate, Italy; 2 Dipartimento di Informatica, Università degli Studi di Milano, 20133 Milan, Italy; massimo.rivolta@unimi.it; 3 Istituto di Sistemi e Tecnologie Industriali Intelligenti per il Manifatturiero Avanzato, Consiglio Nazionale delle Ricerche, 20133 Milan, Italy; alessandro.scano@stiima.cnr.it

## 1. Introduction

The rapid advancement of biomedical sensor technology has revolutionized the field of functional mapping in medicine, offering novel and powerful tools for diagnosis, clinical assessment, and rehabilitation. The ability to collect and analyze various physiological signals, even in real-time, has provided unprecedented insights into the “hidden” functioning of the human body. Biomedical sensors have not only enhanced our understanding of human physiology but have also significantly impacted clinical decision-making, patient management, and the development of personalized medical interventions.

This Special Issue presents a collection of 14 papers that showcase the diverse applications of biomedical sensors in the context of functional mapping. The papers can be grouped into three sections, highlighting their contributions to (i) medical diagnosis, detection and prediction; (ii) neurological and rehabilitation assessment; and (iii) medical applications and monitoring. Together, these papers shed light on the transformative role of biomedical sensors in understanding physiological mechanisms and enhancing healthcare practices.

## 2. Biomedical Sensors for Diagnosis, Detection and Prediction

This section focuses on the application of biomedical sensors for medical diagnosis, detection and prediction. The papers included in this section have a specific focus on the detection of conditions such as COVID-19 and hand osteoarthritis and the prediction of emotions by biosignals. Furthermore, novel approaches based on artificial intelligence and cutting-edge technologies are described.

The paper “COVID-19 Detection Using Photoplethysmography and Neural Networks” [1] presents a groundbreaking approach that utilizes deep learning and raw photoplethysmography signals acquired from a pulse oximeter to identify COVID-19 patients. Achieving an impressive 83.86% accuracy and 84.30% sensitivity in identifying COVID-19 patients, this non-invasive and cost-effective method holds promise for early detection and management of the COVID-19 pandemic, particularly in resource-limited healthcare settings.

In the paper “Toward Early and Objective Hand Osteoarthritis Detection by Using EMG during Grasps” [2], researchers explore the potential of electromyography (EMG) in detecting hand osteoarthritis at an early stage. By studying EMG characteristics during hand grasping tasks, the study provides valuable insights into identifying hand osteoarthritis patients before joint degeneration occurs, enabling timely intervention and improved patient outcomes.

The third paper “Applications of Laser-Induced Fluorescence in Medicine” [3] explores the various medical applications of laser-induced fluorescence (LIF). This highly sensitive spectroscopic method proves valuable in diagnosing and monitoring conditions such as cancer, dental diseases, and fungal infections, offering a versatile tool for medical diagnostics.

Additionally, the paper “Predicting Emotion with Biosignals: A Comparison of Classification and Regression Models for Estimating Valence and Arousal Level Using Wearable Sensors” [4] delves into predicting emotions using biosignals collected via wrist-worn sensors. By comparing different prediction models, the study highlights the effectiveness of regression models, particularly LSTM-based, in estimating emotional valence and arousal levels, enhancing our understanding of human emotions and their applications in healthcare.

## 3. Biomedical Sensors for Neurological and Rehabilitation Assessment

This section explores the applications of biomedical sensors in neurological assessment and rehabilitation. The papers collected in this section describe interesting advancements in the analysis of electroencephalography (EEG), EMG and Near-Infrared Spectroscopy (NIRS) signals for the extraction of biomarkers to characterize individual status in neuromuscular applications that can have a potential impact, for example, on the assessment of rehabilitation effectiveness.

The paper “Reliability of Mental Workload Index Assessed by EEG with Different Electrode Configurations and Signal Pre-Processing Pipelines” [5] evaluates the reproducibility and sensitivity of mental workload assessment from EEG signals using different electrode configurations and pre-processing pipelines. The findings provide valuable insights into developing reliable methods for assessing cognitive tasks, crucial for enhancing human performance in various domains.

In the paper “A Novel Approach for Segment-Length Selection Based on Stationarity to Perform Effective Connectivity Analysis Applied to Resting-State EEG Signals” [6], researchers proposed a novel approach for selecting appropriate segment lengths in EEG-based effective connectivity analysis. By addressing the critical issue of segment-length selection, this method offers valuable insights into studying brain network interactions during resting-state, improving our understanding of brain function and connectivity.

The paper “Reliable Fast (20 Hz) Acquisition Rate by a TD fNIRS Device: Brain Resting-State Oscillation Studies” [7] introduces a high-power setup for multichannel time-domain functional NIRS measurements. This high-speed acquisition method holds potential applications in studying brain resting-state oscillations, providing valuable information for neuroscientific and clinical research.

The paper “Combined Use of EMG and EEG Techniques for Neuromotor Assessment in Rehabilitative Applications: A Systematic Review” [8] presents a systematic review of combined EEG and EMG techniques in neuromotor assessment during rehabilitation. The review highlights the potential of cortico-muscular interactions for improving rehabilitation approaches in patients with impaired locomotor functions, paving the way for innovative rehabilitation strategies.

Next, the paper “Whole-Body Adaptive Functional Electrical Stimulation Kinesitherapy Can Promote the Restoring of Physiological Muscle Synergies for Neurological Patients” [9] introduces a novel treatment approach, Adaptive Functional Electrical Stimulation Kinesitherapy (AFESK™), for neurological patients using whole-body adaptive functional electrical stimulation kinesitherapy. This treatment shows promise in restoring physiological muscle synergies, enhancing motor functionality, and improving rehabilitation outcomes.

Finally, the paper, “Technology Acceptance Model for Exoskeletons for Rehabilitation of the Upper Limbs from Therapists’ Perspectives” [10] addresses the challenges of integrating exoskeleton technology into clinical practice for upper limb rehabilitation. By investigating therapists’ perspectives on exoskeleton acceptability, this study reveals factors influencing their willingness to adopt the technology. The findings suggest that integrating exoskeletons with multi-sensor feedback systems may improve acceptance and facilitate better patient outcomes.

## 4. Biomedical Sensors for Medical Applications and Monitoring

This last section emphasizes the role of biomedical sensors in medical applications and monitoring, describing novel technologies and tools to improve health monitoring in different medical scenarios.

The paper “Towards a Practical Implementation of a Single-Beam All-Optical Non-Zero-Field Magnetic Sensor for Magnetoencephalographic Complexes” [11] introduces a single-beam all-optical two-channel magnetic sensor scheme developed for non-zero-field magnetoencephalography and magnetocardiography applications. This innovative sensor scheme utilizes a single laser beam with time-modulated linear polarization to detect magnetic resonance, providing valuable insights for neurological assessments and diagnostic applications.

The paper “Experimental Assessment of Cuff Pressures on the Walls of a Trachea-Like Model Using Force Sensing Resistors: Insights for Patient Management in Intensive Care Unit Settings” [12] investigates the pressures exerted by endotracheal tube cuffs on the walls of a test bench mimicking the laryngotracheal tract. The study provides valuable insights for patient management in intensive care unit settings, highlighting the need for periodic checks of cuff pressure to prevent pressure-related complications.

Continuing in the realm of prosthetic control, the paper “Questioning Domain Adaptation in Myoelectric Hand Prostheses Control: An Inter- and Intra-Subject Study” [13] delves into the challenges of domain adaptation techniques in myoelectric hand prosthesis control. The results question the conventional approach based on transfer learning and suggest the need for further exploration in this area.

The last paper in this section, “Multi-Scale Evaluation of Sleep Quality Based on Motion Signal from Unobtrusive Device” [14], introduces a multi-scale method for evaluating sleep behavior using motion signals obtained from a pressure bed sensor. The algorithm provides a good correlation between sleep quality measures obtained with polysomnography and pressure bed sensors, offering potential applications for home monitoring of sleep and improving subjects’ awareness of potential sleep disorders.

## 5. Conclusions

The Special Issue *“Biomedical Sensors for Functional Mapping: Techniques, Methods, Experimental and Medical Applications”* presents a comprehensive collection of cutting-edge research in the field of biomedical sensors. The papers cover a wide range of applications, including medical diagnosis and detection, neurological assessment and rehabilitation, and medical monitoring. These advancements pave the way for improved healthcare practices, patient outcomes, and personalized medicine. As biomedical sensor technology continues to evolve, the findings from these research studies hold significant promise in revolutionizing medical practices and addressing complex health challenges, ultimately leading to better human health and well-being.
